# Antimalarial drugs and the prevalence of mental and neurological manifestations: A systematic review and meta-analysis

**DOI:** 10.12688/wellcomeopenres.10658.2

**Published:** 2017-06-02

**Authors:** Mary A. Bitta, Symon M. Kariuki, Clifford Mwita, Samson Gwer, Leah Mwai, Charles R.J.C. Newton

**Affiliations:** 1 KEMRI-Wellcome Trust Research Programme, Centre for Geographic Medicine Research (Coast), Kilifi, Kenya; 2 Department of Surgery, Thika Level 5 Hospital, Thika, Kenya; 3 Joanna Briggs Institute (JBI) Affiliate Centre for Evidence-Based Healthcare in Kenya, Clinical Research Evidence Synthesis and Translation Unit, Afya Research Africa, Nairobi, Kenya; 4 Department of Medical Physiology, School of Medicine, Kenyatta University, Nairobi, Kenya; 5 Department of Psychiatry, University of Oxford, Oxford, UK

**Keywords:** Antimalarial drugs, Mental and neurological manifestations, Toxicity, Systematic review, Meta-Analysis

## Abstract

**Background:** Antimalarial drugs affect the central nervous system, but it is difficult to differentiate the effect of these drugs from that of the malaria illness. We conducted a systematic review to determine the association between anti-malarial drugs and mental and neurological impairment in humans. **Methods:** We systematically searched online databases, including Medline/PubMed, PsychoInfo, and Embase, for articles published up to 14th July 2016. Pooled prevalence, heterogeneity and factors associated with prevalence of mental and neurological manifestations were determined using meta-analytic techniques. **Results:** Of the 2,349 records identified in the initial search, 51 human studies met the eligibility criteria. The median pooled prevalence range of mental and neurological manifestations associated with antimalarial drugs ranged from 0.7% (dapsone) to 48.3% (minocycline) across all studies, while it ranged from 0.6% (pyrimethamine) to 42.7% (amodiaquine) during treatment of acute malaria, and 0.7% (primaquine/dapsone) to 55.0% (sulfadoxine) during prophylaxis. Pooled prevalence of mental and neurological manifestations across all studies was associated with an increased number of antimalarial drugs (prevalence ratio= 5.51 (95%CI, 1.05-29.04); P=0.045) in a meta-regression analysis. Headaches (15%) and dizziness (14%) were the most common mental and neurological manifestations across all studies. Of individual antimalarial drugs still on the market, mental and neurological manifestations were most common with the use of sulphadoxine (55%) for prophylaxis studies and amodiaquine (42.7%) for acute malaria studies. Mefloquine affected more domains of mental and neurological manifestations than any other antimalarial drug. **Conclusions:** Antimalarial drugs, particularly those used for prophylaxis, may be associated with mental and neurological manifestations, and the number of antimalarial drugs taken determines the association. Mental and neurological manifestations should be assessed following the use of antimalarial drugs.

## Introduction

Over 3.2 billion people in the world are at risk of malaria (Malaria fact sheet, World Health Organization) and a wide range of antimalarial drugs is used to prevent and treat malaria. Malaria continues to be a major cause of morbidity and mortality, but both have declined with the introduction of effective anti-malarial drugs malaria (Malaria fact sheet, World Health Organization). Mental and neurological manifestations are common in patients with malaria, particularly children admitted to hospital with falciparum malaria in Africa ^1, 2^. Survivors of severe malaria develop a wide range of neuro-cognitive sequelae, including epilepsy, language deficits, motor and sensory deficits, and other neurobehavioral difficulties ^3, 4^. Antimalarial drugs are thought to have significant mental and neurological manifestations ^5– 7^. Antimalarials prescribed to prevent malaria are associated with mental and neurological manifestations, some of which are similar to the manifestations seen in acute malaria ^8– 10^. Therefore, the neuro-cognitive and behavioural sequelae observed after malaria may be related to the underlying malarial illness or the antimalarial drugs. Teratogenicity is reported after the use of antimalarial drugs in pregnancy, but mental and neurological damage is not extensively studied ^11^.

Mental and neurological manifestations of antimalarial drugs are observed in both animals and humans ^8, 12– 18^. Animal studies identify potential mechanisms of mental and neurological manifestations and the parts of the central nervous system (CNS) affected. To date, there have been no human studies attempting to explore the role that drugs play in causing mental and neurological manifestations after accounting for the malarial illness, although some studies have acknowledged that malaria illness alone cannot explain neuro-cognitive and behavioural sequelae observed after treatment ^4^. Reports have highlighted severe neuropsychiatric reactions after use of mefloquine for prophylaxis ^19, 20^, but properly designed studies are required to quantify and clarify the extent of these manifestations. Studying the use of antimalarial drugs for prophylaxis can help to estimate the prevalence of mental and neurological manifestations in non-infected subjects, and compare with the prevalence observed in patients with malaria to understand if antimalarial drugs add to mental and neurological manifestations.

We conducted a systematic review and meta-analysis of the published literature on mental and neurological manifestations associated with antimalarial drugs, and reported the findings according to the PRISMA guidelines ^21^. We estimated the pooled overall prevalence of mental and neurological outcomes among the human studies identified and examined if prevalence differed by type and number of antimalarial drugs used. We also investigated and quantified the sources of heterogeneity between the studies and attempted to identify the factors explaining the variation in prevalence of mental and neurological outcomes.

## Methods

### Information sources

We searched the following online databases systematically: MEDLINE, EMBASE, CINHL, PsycINFO, Central Registration for Clinical Trials, Open Grey Database, Canadian Agency for Drugs and Technologies in Health, Directory of Open-Access Repository, World Cat database, and Web of Science. Reference lists of identified articles were also searched for relevant titles and these were in turn searched online. All authors contributed to the search strategy. Consensus was used to set the selection criteria according to recommendations ^22^.

### Search strategies

An initial limited search of MEDLINE, COCHRANE LIBRARY and EMBASE was undertaken followed by analysis of the text words contained in the title and abstract, and of the index terms used to describe articles. Combined text words and Medical Subject Headings (MeSH) terminology were used in addition to the two main search terms/facets [Mental and neurological and Antimalarial Drugs] ( Supplementary Table 1). Boolean operators, such as “AND” and “OR”, were used to combine search terms as necessary. Truncation, wildcard, adjacent searching, and floating subheadings were also used to increase the sensitivity of the results in unpublished data, where necessary. The construction of search terms followed the recommendations by the National Health Service Centre for Reviews and Disseminations.

### Inclusion and exclusion criteria

We included studies that met the following criteria: (i) use of an antimalarial agent ( Table 1) (either as a prophylactic drug or as treatment for malaria or another illness); and (ii) report of mental and neurological symptoms, including psychiatric disorders, cognitive impairments, sensory problems, and seizures (during or after using the antimalarial) ( Table 2). We also included studies reporting foetal teratogenicity following use of antimalarial drug in pregnancy. Only empirical studies were considered for the main analysis, while case series/reports studies were excluded because their findings cannot be generalized. There was no restriction on age of the participants or on the study settings.

**Table 1.** Classification of antimalarial drugs.

Class	Drugs
4-Aminoquinolines	Chloroquine, amodiaquine, hydroxychloroquine
8-Aminoquinoline	Primaquine, pamaquine, pentaquine, isopentaquine
4-quinolinemethanols	Quinine, quinidine, mefloquine
Phenanthrene methanol	Halofantrine
Artemisinin derivatives	Artemisinin, artemether, artesunate, arteether
Antimetabolites	Proguanil, pyrimethamine, atovaquone, dapsone
Antibiotics	Tetracycline, doxycycline, minocycline
Diaminopyridines	Pyrimethamine

**Table 2.** Classification of neurological manifestations.

Category	Specific symptoms
Psychiatric disorders	Suicidality, violence, hallucinations, delusions, psychosis, depression, phobias, anxiety, anorexia
Mild neurological perturbations	Stupor, dizziness, fainting, confusion
Motor impairment	Motor impairments, ataxia
Sleep disturbances	Nightmares, vivid dreams, insomnia, sleep pattern disturbance
Personality changes	Mood changes, altered esteem, personality changes
Sensory impairments	Peripheral neuropathy, anorexia, paraesthesia
Seizures	Convulsions, seizures
Headache	Headache
Hearing and balance	Hearing loss, tinnitus, vertigo
Visual	Blurred vision, diplopia, loss of vision
Cognition	Altered memory, concentration problems, speech problems

Eligible English articles were considered and articles in French, Dutch, Chinese, Hebrew, Spanish, and German were also retrieved, translated and reviewed for eligibility. Unpublished work and proceedings from scientific conferences were included in the review if they fulfilled the criteria above. There were no restrictions on dates of earliest possible publications, but articles published up to 14 ^th^ July 2016, which was the last search date, were included. Excluded from the analysis were commentaries and conference abstracts without full length and duplicate publications, as were studies of special duplicate populations ^21^.

### Data extraction

Data was extracted into a Microsoft Excel spreadsheet with a list of variables ( Supplementary Table 2) determined *a priori* by the authors. The template was piloted on ten randomly selected studies that satisfied the inclusion criteria. The extraction was performed manually included a two-stage process: first, a determination of eligibility based on titles and abstracts; second, determination of eligibility after reviewing the full texts.

Eligibility assessment was performed independently in a standardized manner by MAB under the guidance of SMK and CRN. All articles were reviewed by at least two authors. Disagreements between reviewers were resolved by consensus. Follow-up time was defined as the number of days between administration of the antimalarial agent and the appearance of the mental and neurological symptoms.

**Figure 1.**
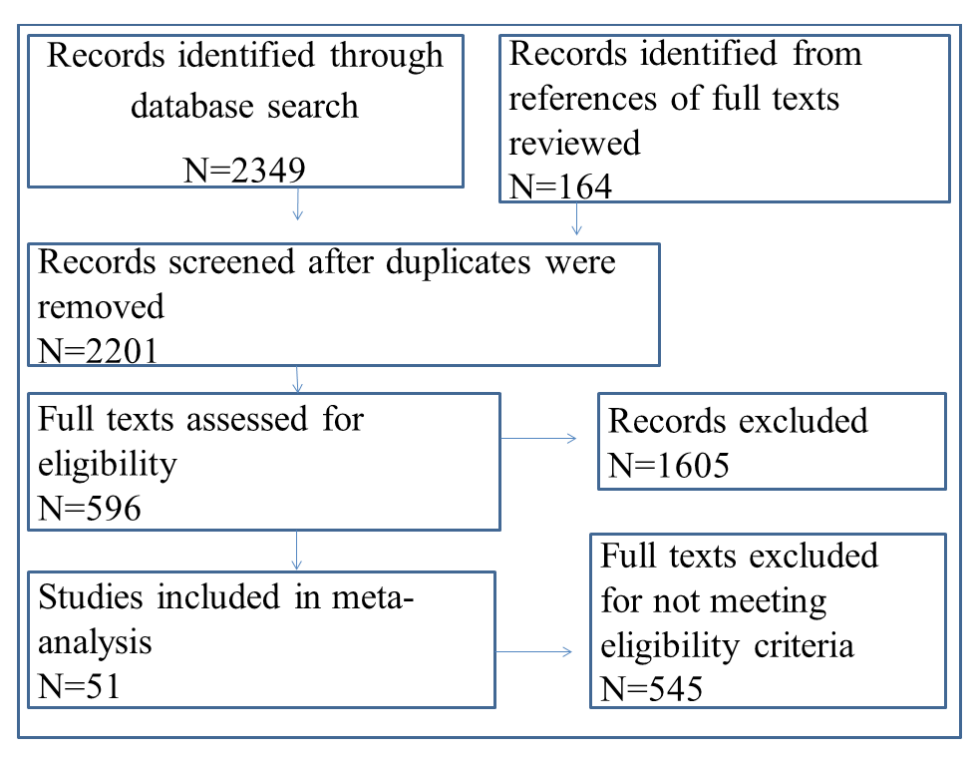
Study selection process.

Where overall prevalence was not reported, but an *n* (number of people reporting a specific symptom) was assigned for each mental and neurological symptom, we took the symptom for which *n* had the highest value and calculated the overall prevalence using the formula ( *n*/N)*100; where N= total sample size of the study. The assumption was that the symptoms were not mutually exclusive. The extracted information contained Population, Interventions/treatment, Comparison groups, and Outcomes (PICO) ^21^. Of the 120 studies in languages other than English, retrieval and translation was only possible for 12 full articles. Figure 1 illustrates the selection process ^23^.

### Classification of mental and neurological symptoms

Anti-malarial drug effects of the eight classes of antimalarial drugs were studied under ( Table 1). Specific mental and neurological symptoms were classified into 12 categories of related symptoms ( Table 2).

### Critical appraisal of studies included in meta-analysis

The quality of all observational studies that met the inclusion criteria was investigated using the The Joanna Briggs Institute Prevalence Critical Appraisal Tool ^24^. The tool is a ten question questionnaire with four possible responses: yes, no, unclear or not applicable. Scores of quality were calculated as a percentage, with ten as the denominator unless a section was marked as ‘not applicable’, in which case we excluded that section from the total quality score. This was done to avoid downgrading the total score of quality by a domain that does not apply to that study. Each positive (yes) response to a domain was equal to one point, whereas a negative response (no/unclear) attracted no point. For experimental studies, the Grading of Recommendations Assessment, Development, and Evaluation (GRADE) system was used ^25^. Studies which fulfilled >80% of the criteria for quality were included in this review.

### Statistical analysis

**Figure 2.**
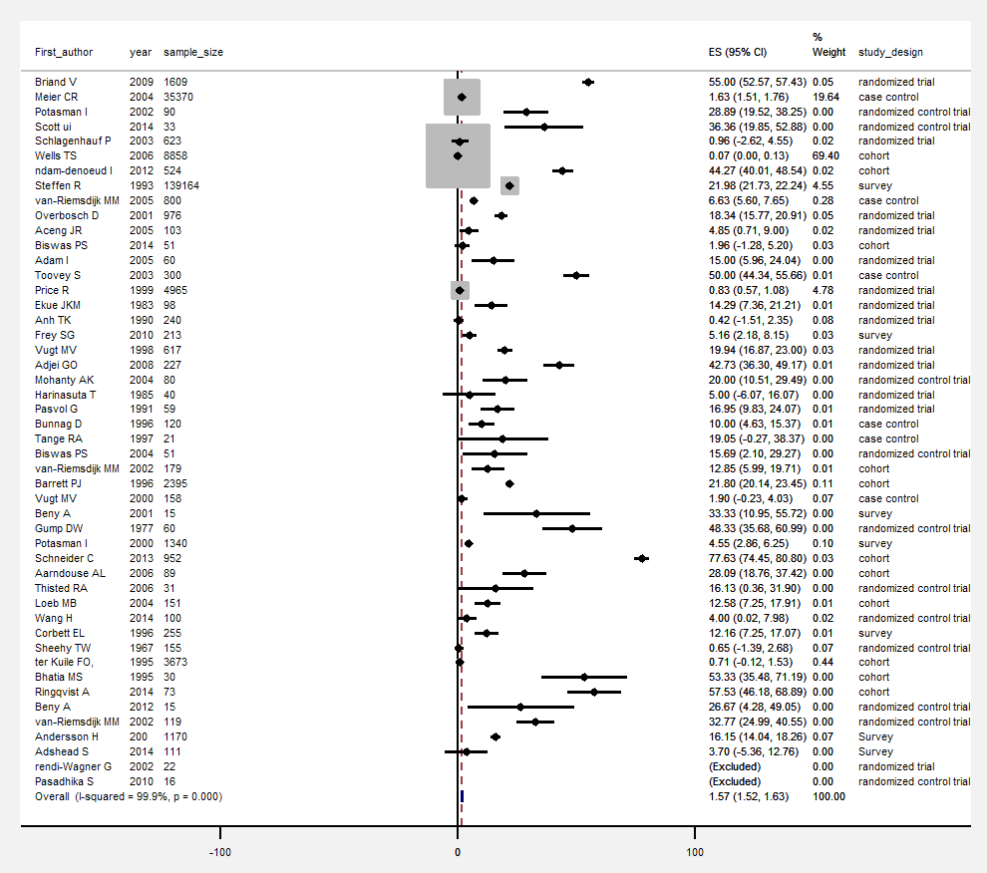
Forest plot of the prevalence of mental and neurological manifestations in studies included in the meta-analysis.

We computed crude median prevalence of mental and neurological manifestations expressed per 100 subjects or as a percentage and the corresponding interquartile ranges (IQR). Prevalence of mental and neurological manifestations was determined for human studies only. The 95% confidence interval (95%CI) for each study’s prevalence of mental and neurological outcomes were calculated using the formula: $p\pm [1.96\;\times \;\sqrt{p(100\;-\;p)}]/N$; where p is the prevalence as a percentage and N is the sample size ^26^. For unweighted pooled median prevalence of mental and neurological manifestations, we fitted a random effect model to the individual study prevalence estimates and their corresponding 95%CI using STATA version 13.1 (Stata Corp, Texas, USA). Random effect models allow effect estimates to vary across the studies. The pooled estimates from this model and their corresponding 95%CIs were obtained on the original prevalence scale. These estimates were also summarised in a forest plot ( Figure 2). Comparison of the spectrum of mental and neurological manifestations and/or their severities across treatment groups was done with Persons Chi-square test or Fishers exact test, where appropriate. Comparison of prevalence between those on antimalarial drugs and controls not on antimalarial drugs was done using Pearson’s Chi-square test.

**Figure 3.**
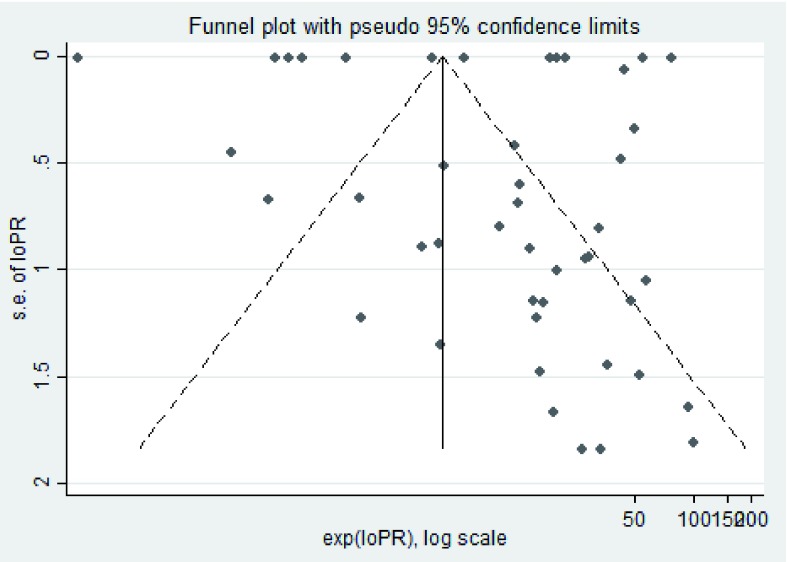
A funnel plot of bias of the selected studies.

The Cochrane Q-statistic was used to test the null hypothesis that the prevalence of mental and neurological manifestations was uniform across the studies. The degree of heterogeneity (I ^2^) of the pooled estimates was derived from the random effect models as a function of the Q statistics and degrees of freedom, expressed as a percentage ([Q-df/Q] × 100). We further investigated the contribution of factors, such as age, study design and malaria status, to the variation in the documented prevalence of mental and neurological outcomes. This was implemented by fitting two random effect meta-regression models, one as a null model without the covariates of interest and another with the variables of interest, both models with the documented prevalence as the dependent variable and the associated standard errors specified. The proportion of variation explained by the covariates studied was determined by dividing the difference in components of variance between the two models (τ0 ^2^ − τ ^2^) by the variance in the null model (τ0 ^2^). Where two variables showed strong multi-collinearity, one was picked at random for inclusion in the multivariable model. Reporting and publication ( Figure 3) bias were examined in STATA using funnel plots.

## Results

### Search results and study characteristics

The initial search yielded 2349 results, of which 596 were retained for full review based on title and abstract examination ( Figure 1). After full text review, we excluded 545 articles in the quantitative analysis: articles in foreign languages for which translation could not be obtained (N=108); articles that did not mention neurotoxic outcomes (N=341); reviews (N=16) and case reports (N=80). A total of 51 studies with a reporting on 205,175 subjects were retained. The study characteristics are defined in Table 3.

**Table 3.** Description of study characteristics.

First author (Ref)	Year	Country	Design	Sample size	Malaria study	Region	Children/ Adult	Sex (% female)
Sjowall J ^27^	2012	Sweden	Randomized control trial	15	No	High income	Adult	40.0
Scott UI ^28^	2014	USA	Randomized control trial	33	No	High income	Adult	36.4
Frey SG ^9^	2010	Cameroon	Survey	213	Yes	Low income	Child	51.2
Wells TS ^29^	2006	Japan	Cohort	8858	No	High income	Adult	0.0
Pasadhika S ^10^	2010	USA	Randomized control trial	16	No	High income	Adult	100.0
Van-riemsdijk MM ^30^	2004	Netherlands	Cohort	151	No	High income	Adult	57.1
Bhatia MS ^31^	1995	India	Cohort	30	No	Low income	Adult	Absent
Potasman I ^32^	2000	Israel	Survey	1340	No	High income	Adult	54.7
Ter Kuile FO ^33^	1995	Thailand	Cohort	3673	No	High income	Adult	37.6
Corbett El ^34^	1996	Britain	Survey	255	No	High income	Adult	Absent
Barrett PJ ^35^	1996	Britain	Cohort	2395	No	High income	Adult	58.1
Beny A ^5^	2001	Israel	Survey	15	No	High income	Adult	33.3
Gump DW ^36^	1977	USA	Randomized control trial	60	No	High income	Adult	100.0
Wang H ^37^	2014	China	Randomized control trial	100	No	High income	Adult	53.0
Schneider C ^38^	2013	Switzerland	Cohort	952	No	High income	Adult	66.5
Biswas PS ^39^	2014	India	Cohort	51	No	Low income	Adult	50.0
Sheehy TW ^40^	1967	Vietnam	Randomized control trial	155	No	Low income	Adult	0.0
Thisted RA ^6^	2006	USA	Randomized control trial	31	No	High income	Adult	17.0
Aarnoudse Al ^41^	2006	Absent	Cohort	89	No	Absent	Adult	46.1
Van-riemsdijk MM ^42^	2002	Netherlands	Cohort	179	No	High income	Adult	46.9
Bhatia MS ^31^	1994	India	Randomized control trial	30	No	High income	Adult	Absent
Ringqvist A ^43^	2014	Denmark	Cohort	73	No	High income	Adult	54.8
Loeb MB ^44^	2004	Canada	Randomized control trial	51	No	High income	Adult	58.8
Held TH ^45^	1991	Germany	Randomized control trial	20	No	High income	Adult	Absent
Adjei GO ^46^	2008	Ghana	Randomized trial	227	Yes	High income	Child	47.6
Briand V ^47^	2009	Benin	Randomized trial	1609	No	Low income	Adult	100
Vugt MV ^48^	2000	Thailand	Case control	158	No	High income	Mixed	29.1
Aceng JR ^49^	2005	Uganda	Randomized trial	103	Yes	Low income	Child	Absent
Adam I ^50^	2005	Sudan	Randomized trial	60	Yes	Low income	Adult	56.7
Toovey S ^51^	2003	Mozambique	Case control	300	Yes	Low income	Adult	1.3
Tange RA ^52^	1997	Netherlands	Case control	21	Yes	High income	Adult	38.1
Pasvol G ^53^	1991	Kenya	Randomized trial	59	Yes	Low income	Child	Absent
Schlagenhauf P ^54^	2003	Multiple	Randomized trial	623	No	High income	Adult	Absent
Vugt MV ^55^	1998	Thailand	Randomized trial	617	Yes	High income	Mixed	31.8
Ekue JMK ^56^	1983	Zambia	Randomized trial	98	Yes	Low income	Mixed	0
Price R ^57^	1999	Thailand	Randomized trial	4965	Yes	High income	Mixed	43.7
Overbosch D ^58^	2001	Multiple	Randomized trial	976	No	Low income	Mixed	45
Anh TK ^59^	1990	Vietnam	Randomized trial	240	Yes	Low income	Mixed	0
Bunnag D ^60^	1996	Thailand	Comparative study	120	Yes	High income	Adult	Absent
Ndam-Denoeud L ^61^	2012	Benin	Cohort	524	No	Low income	Adult	0
Harinasuta T ^62^	1985	Thailand	Randomized trial	40	Yes	High income	Mixed	40
Mohanty AK ^63^	2004	India	Randomized control trial	80	Yes	Low income	Child	42
Potasman I ^64^	2002	Israel	Randomized control trial	90	No	High income	Adult	54.6
Rendi-Wagner P ^65^	2002	Austria	Randomized trial	22	No	High income	Adult	52.5
Steffen R ^66^	1993	Europe	Survey	139164	No	High income	Mixed	70
Van-riemsdijk MM ^67^	2005	Netherlands	Case control study	800	No	High income	Adult	Absent
Meier CR ^68^	2004	UK	Case control study	35370	No	High income	Adult	37.8
Van Riemsdijk MM ^7^	2002	Netherlands	Randomized control trial	119	No	High income	Adult	Absent
Andersson H ^69^	2008	Sweden	Survey	1170	No	High income	Adult	4.8
Adshead S ^70^	2014	UK	Survey	111	No	High income	Adult	87.5

There were 5 studies on children (<18 years) with a total sample size of 682 (3.4%). The remaining studies were either studies on adults or mixed populations with a population of 205,175. The male and female sex ratio in the studies was well balanced (P=0.357). The median follow up time was 8 days (IQR, 3–28) for the studies that reported follow up data.

### Estimates of overall prevalence and heterogeneity

Of the 51 eligible studies, 48 (94.9%) reported a prevalence of at least one category of mental and neurological outcomes. The estimated range of pooled prevalence of mental and neurological manifestation following antimalarial drug use from the random effect models of all the studies was between 0.7% (95%CI 0.62–1.91) for primaquine and dapsone users to 48.3% (95%CI 35.7–61.0) for minocycline users. The random effect model of the pooled prevalence across all human studies was associated with a very high between-study heterogeneity (Q=10.94, I ^2^=97.8%). There were only two studies (N=229) in which controls were persons who were not taking any antimalarial drugs ^48, 51^. In these two studies, the average prevalence of mental and neurological manifestations was 3.0%.

### Factors explaining the variation in documented overall prevalence

Several factors were assessed in the univariable analysis of human studies, and five appeared to explain the highest variation in the documented median prevalence, but none reached statistical significance level of P<0.05 ( Table 4). In the multivariable meta-regression analysis, the number of drugs used was independently associated with the prevalence of mental and neurological outcomes (Prevalence ratio=5.51 [95% CI, 1.05–29.04], P=0.045). Other factors, such as being a child and having an acute malarial illness, were not associated with variation in prevalence of mental and neurological outcomes. The factors investigated in the multivariable analysis explained 14.1% variability of the prevalence across all human studies. In the multivariable linear regression model, there was no evidence for interaction between malaria illness and the number of drugs in explaining the variation in prevalence of symptoms (interaction parameter: beta co-efficient=2.38 [95%CI, 0.15–37.32; P=0.503]).

**Table 4.** Heterogeneity and associated factors.

Factor	Univariable analysis			Multivariable analysis	
	Prevalence ratio (95%CI)	P value	Heterogeneity (%)	Prevalence ratio (95%CI)	P value
Year of publication	1.04 (0.99–1.1)	0.125	4.3	0.74 (0.39–1.41)	0.789
Study design	0.91 (0.71–1.15)	0.407	1.2	0.84 (0.45–1.56)	0.372
Malaria *vs* prophylaxis study	0.99 (0.21–4.71)	0.988	23.4	2.38 (0.15–37.32)	0.663
Paediatric study	1.05 (0.18–6.02)	0.955	2.9	0.87 (0.04–19.73)	0.945
Number of drugs	1.11 (0.62–2.00)	0.719	0.9	5.51 (1.05–29.04)	0.045

### Pooled prevalence of mental and neurological manifestations of individual antimalarial drugs

The highest pooled prevalence of mental and neurological manifestations in the prophylaxis group was reported in those on sulphadoxine (55.0%; 95%CI 52.6–57.4) followed by minocycline (48.3%; 95%CI 35.7–61.0). In the treatment groups, patients receiving amodiaquine reported the highest prevalence (42.7%; 95%CI 36.3–49.2) followed by those on lumefantrine (29.5%; 95%CI 27.0–32.0) ( Table 5). The lowest overall prevalence was reported by dapsone and primaquine users (0.7%; 95%CI 0.6–1.9), while for prophylaxis it was 0.6% (95%CI 0.2–1.4; pyrimethamine) and for malaria studies it was 0.7% (95%CI 0.6–1.9; quinine, primaquine and dapsone).

**Table 5.** Overall pooled prevalence of mental and neurological manifestations for individual drugs.

Antimalarial drug	Estimated percentage pooled median prevalence with corresponding 95%CI	Median overall prevalence in prophylaxis studies (95%CI)	Median overall prevalence in malaria studies c(95%CI)
Amodiaquine	42.7 (36.3–49.2)	-	42.7 (36.3–49.2)
Artemether	1.1 (0.9–1.4)	2.4 (0.5–4.3)	1.1 (0.9–1.4)
Artesunate	1.1 (0.9–1.3)	1.9 (0.2–4.0)	1.1 (0.8–1.3)
Atovaquone	8.0 (7.3–8.7)	8.7 (8.0–9.4)	8.0 (7.3–8.7)
Chloroquine	7.1 (7.0–7.2)	7.1 (7.0–7.2)	4.9 (1.9–7.8)
Dapsone	0.7 (0.6–1.9)	0.7 (0.6–1.9)	-
Doxycycline	1.6 (1.5–1.8)	1.6 (1.5–1.8)	-
Lumefantrine	29.5 (27.0–32.0)	-	29.5 (27.0–32.0)
Mefloquine	1.9 (1.6–2.1)	25.0 (23.8–26.3)	1.0 (0.7–1.2)
Minocycline	48.3 (35.7–61.0)	48.3 (35.7–61.0)	-
Primaquine	0.7 (0.6–1.9)	0.6 (0.6–1.9)	-
Proguanil	7.3 (7.2–7.4)	7.3 (7.2–7.4)	-
Pyrimethamine	20.8 (20.6–21.0)	22.3 (22.0–22.5)	0.6 (0.2–1.4)
Quinidine	16.1 (3.2–29.1)	16.1 (3.2–29.1)	-
Quinine	1.7 (0.5–2.9)	0.7 (0.6–1.9)	9.3 (5.9–12.7)
Sulfadoxine	6.0 (5.2–6.8)	55.0 (52.6–57.4)	0.6 (0.1–1.4)

- No observations

### Spectrum of mental and neurological effects

For all the studies that reported the number of subjects with specific mental and neurological symptoms (N=205,120), headache was the most frequent symptom (N=30,726; 15.0%), followed by dizziness (N=28,626; 14.0%); neither being mutually exclusive of other mental and neurological outcomes. For studies of acute treatment of malaria, the commonest mental and neurological manifestations were hearing and balance problems (N=184; 2.6%). For prophylactic studies, the commonest manifestations were headaches (N=30,709; 15.5%) and dizziness (N=28,472; 14.4%).

### Individual drugs and domains of mental and neurological outcomes

***(a) Malaria treatment studies***. Mefloquine and quinine were associated with mental and neurological manifestations in more of the domains (6 out of 10) investigated than any other antimalarial drug. Mild neurological perturbations were the most commonly reported symptom in all individual drugs studied, except amodiaquine, with the highest prevalence being in lumefantrine (10.8%). Cognition problems were infrequently reported in malaria treatment studies ( Table 6).

**Table 6.** Mental and neurological outcomes for individual drugs by malaria treatment studies. -No observations.

Drug	Psychiatric/ behavioural	Mild neurological perturbations	Motor problems	Sleep pattern disturbances	Personality changes	Seizures	Headache	Hearing & balance problems	Visual problems	Cognition problems
**Mefloquine,** **N=6293 (%)**	2 (0.03)	136 (2.16)	40 (0.64)	114 (1.81)	1 (0.02)	1 (0.02)	-	-	-	-
**Chloroquine,** **N=189 (%)**	2 (1.06)	14 (7.41)	-	-	1 (0.53)	1 (0.53)	-	-	-	-
**Sulfadoxine,** **N=340 (%)**	2 (0.59)	9 (2.65)	-	-	-	-	1 (0.29)	-	-	-
**Pyrimethamine,** **N=340 (%)**	2 (0.59)	9 (2.65)	-	-	-	-	1 (0.29)	-	-	-
**Artemether,** **N=6332 (%)**	-	123 (1.94)	40 (0.63)	114 (1.80)	-	-	-	150 (2.37)	-	-
**Artesunate,** **N=6282 (%)**	-	136 (2.16)	40 (0.64)	114 (1.81)	-	-	16 (0.25)	8 (0.13)	1 (0.02)	-
**Quinine, N=263** **(%)**	-	8 (3.04)	10 (3.80)	-	-	5 (1.90)	16 (6.08)	34 (12.93)	1 (0.38)	-
**Lumefantrine,** **N=1144 (%)**	-	123 (10.75)	40 (3.50)	114 (9.97)	-	-	-	150 (13.11)	-	-
**Amodiaquine,** **N=227 (%)**	**-**	**-**	**-**	**-**	**-**	**-**	**-**	**-**	**-**	**-**

***(b) Prophylaxis studies***. Mefloquine was associated with mental and neurological manifestations in 8 out of 10 domains investigated, lacking prevalence reports in only motor impairments and seizures. Chloroquine and proguanil reported 6 out of the 10 domains each ( Table 6). Psychiatric/behavioural problems were reported in all the drugs examined except in quinidine and minocycline, with the highest prevalence being in atovaquone (30.7%) followed by pyrimethamine (4.7%). The lowest prevalence of psychiatric symptoms was in sulfadoxine users (0.1%). There were no reports of seizures and/or motor impairments in groups using antimalarial drugs for prophylaxis. The prevalence of mental and neurological manifestations did not differ across categories of dosage (Χ ^2^=4.65, P=0.460). Table 7 summarizes these findings.

**Table 7.** Mental and neurological outcomes for individual drugs for prophylaxis studies. - No observations.

Drug	Psychiatric/ behavioural	Mild neurological perturbations	Motor problems	Sleep pattern disturbances	Personality changes	Seizures	Headache	Hearing & balance problems	Visual problems	Cognition problems
**Atovaquone,** **N=2670 (%)**	819 (30.67)	54 (2.02)	-	179 (6.70)	-	-	51 (1.91)	-	24 (0.90)	-
**Mefloquine,** **N=197959 (%)**	8146 (4.11)	28699 (14.50)	-	23630 (11.94)	9 (0.00)	-	30697 (15.51)	4 (0.00)	14321 (7.235)	53 (0.03)
**Primaquine,** **N=155 (%)**	1 (0.65)	-	-	-	-	-	-	-		-
**Pyrimethamine,** **N=140773 (%)**	6600 (4.69)	28094 (19.96)	-	23280 (16.54)	-	-	30593 (21.73)	-	14297 (10.16)	-
**Chloroquine,** **N=180314 (%)**	7985 (4.43)	28140 (15.61)	-	22456 (12.45)	-	-	30593 (16.97)	-	14297 (7.93)	25 (0.01)
**Proguanil,** **N=180571 (%)**	8060 (4.46)	28194 (15.61)	-	22635 (12.54)	-	-	30644 (16.97)	-	14321 (7.93)	25 (0.01)
**Sulfadoxine,** **N=1609 (%)**	1 (0.06)	-	-	885 (55.00)	-	-	-	-	-	-
**Quinine, N=160** **(%)**	1(0.63)	-	-	-	-	-	-	-	-	-
**Dapsone,** **N=155 (%)**	1 (0.65)	-	-	-	-	-	-	-	-	-
**Doxycycline,** **N=36077 (%)**	578 (1.60)	-	-	8 (0.02)	-	-	-	-	-	-
**Quinidine,** **N=31(%)**	-	5 (16.13)	-	3 (9.68)	-	-	4 (12.90)	1 (3.23)	-	-
**Minocycline,** **N=120 (%)**	-	-	-	-	58 (48.33)	-	44 (36.67)	6 (5.00)	-	48 (40.00)

## Discussion

The pooled estimates from this study show that the prevalence of mental and neurological manifestations differ with antimalarial drugs, as well as malaria status, of individuals using these drugs. The range of overall prevalence is higher in the absence of acute malaria (0.6–42.7% *vs* 0.7–55.0%). Similar to previous reports ^71^, minocycline had the highest prevalence of mental and neurological outcomes (48.3%) and artesunate had the lowest (1.1%). Headaches and dizziness are the most frequent manifestations, and symptoms for psychiatric disorders and cognitive impairment were common with malaria prophylaxis. The number of antimalarial drugs used independently explained the variation in documented overall prevalence. While sulphadoxine and minocycline contributed the highest prevalence of mental and neurological manifestations, mefloquine affected the most domains assessed. These results are based on few human studies (N=51) out of 2,349 abstracts initially identified, suggesting a significant research gap with regards to evaluation of antimalarial drugs for mental and neurological outcomes in humans.

### Prevalence and heterogeneity

The pooled prevalence of mental and neurological manifestations is robust and accounts for heterogeneity between studies, unlike descriptive median estimates, which would otherwise underestimate the true prevalence. The pooled estimates compare favourably with those of some randomised studies, although these studies focused on fewer drugs ^37, 49^. Headaches and dizziness were the most common symptoms, and particularly those on prophylactic treatment, and are often asked for or assessed during studies. Mental and neurological manifestations of the drugs were evaluated in the short-term for most studies (median follow-up, 10 days; IQR, 5–21 days), so this may underestimate prevalence of conditions, such as epilepsy, which take time to develop following neurotoxicity ^65, 72, 73^. This may explain why some domains, such as seizures and motor impairments, were infrequently documented following prophylaxis. There were no reports of cognition problems in malaria studies probably because neurocognition data during the acute phase of malaria may go undocumented due to misattribution of poor cognition to malaria disease, rather than the drugs used to treat the disease, or perhaps because neuropsychological tests are performed following recovery from the episode of acute malaria. The heterogeneity was greater than 70%, usually considered as the proportion attributable to between studies heterogeneity. The excess heterogeneity may be related to bias from publication, reporting and selection, as supported by some studies plotting outside the funnel outline in the meta-funnel analysis as shown in Figure 3.

Most negative studies may be unpublished, since majority of the eligible studies reported at least one domain of mental and neurological manifestations. There was between study variations in the methods of assessment of mental and neurological manifestations which may have contributed to the heterogeneity of pooled estimates observed during the analysis. This informed our decision to apply the random effect model rather than the fixed effect model during analysis.

### Factors explaining variation in prevalence of mental and neurological manifestations

We investigated the contribution of five factors (decided by the authors *a priori*) to the variation in prevalence of mental and neurological manifestations, and found that an increasing number of drug combinations were associated with mental and neurological manifestations. The five factors investigated only explained 14% of the variability in prevalence, and it is possible the prevalence is in part dependent upon other factors unreported in the included studies. These factors may include levels of endemicity of malaria in the study sites and individual factors such as resistance to malaria, which may vary across the eligible studies. The random effects model that we applied for the meta-analysis allowed effect estimates of mental and neurological manifestations to vary across the sites. While the World Health Organization (WHO) recommends a maximum of two drugs in combination therapies aimed at reducing development of resistance to newer drugs, such as artemesinin derivatives (WHO guidelines for malaria treatment), three drug combinations were common (30%) in many studies identified by this review. Although combinations of antimalarials are used to increase efficacy, prevent transmission and reduce resistance, such combinations may increase levels of mental and neurological manifestations, probably from cumulative toxicity of individual drugs. The number of drugs may also be a surrogate marker of possible neurotoxcity, since the two were highly correlated. There was no evidence of interaction between the number of drugs and malaria status/illness, with regard to prevalence of mental and neurological manifestations. Age group was not associated with prevalence of mental and neurological manifestations, although there were significantly fewer children compared to adults in this analysis, yet children bear the brunt for malaria morbidity and mortality in Africa. Ethical considerations may in part explain fewer children participants in the studies, as early-phase clinical trials usually exclude children. The lack of association with year of study may highlight lack of new studies in recent years compared to earlier years, justifying the need for recognition of the problem through conduct of more future studies.

### Mental and neurological manifestations and individual antimalarial drugs

For prophylactic studies, sulfadoxine and minocycline were associated with the highest prevalence of mental and neurological manifestations. For acute malaria studies, hydroxychloroquine was associated with the highest prevalence of mental neurological manifestations. This finding is important as some of these drugs are routinely being used for the treatment of malaria according to WHO recommendations in the guidelines for the treatment of malaria ^72, 74, 75^. Antibiotics, such as doxycycline and tetracycline, which were associated with some mental and neurological manifestations, are often combined with other drugs in 2 ^nd^ line regimens for uncomplicated malaria ^76, 77^, while clindamycin is used during pregnancy ^78, 79^. However, it is important to note that data provided on minocycline are based on one study only and, as Dr Grabias and Dr. Remington noted in their reviews on psychiatric effects of malaria and antimalarials, psychiatric reports following tetracycline use in malaria is almost non-existent ^74, 75^. Artemisinin derivatives are the drugs of choice for malarial treatment when combined with other therapies. Similarly, diaminopyridines, such as pyrimethamine, are combined with artesunate for first line treatment, and are used in pregnancy and prophylaxis (Guidelines for the treatment of malaria, WHO). There is however lack of sufficient studies on their safety to the foetal brain. Some drugs, such as hydroxychloroquine, are no longer routinely recommended by the WHO for use in the management of malaria ^80^, although they are still widely used in the rheumatology community ^81^. Although drugs, such as minocycline, are not in routinely used as an antimalarial, they are still used for conditions, such as acne, for which patients on the drug may benefit from evaluation of mental and neurological status. The low prevalence of mental and neurological manifestations with artemether and artesunate are reassuring, since these are the mainstay drugs for the management of falciparum malaria. Also no prospective studies have examined mental and neurological outcomes of artemisinin derivate use alone ^75^. Reassuringly, very low frequencies were observed for specific domains of neurological manifestation supporting the safety profile of artemether and artesunate. It is however worth noting that this conclusion may be biased since artemether and artesunate are recent drugs for which adverse events are yet to be exhaustively studied. Mefloquine toxicity has been the subject of many case reports ^20, 82– 85^. Our review however found a relatively low prevalence of mefloquine toxicity. Also, unlike a study published by Weinke and colleagues ^86^, our study observed higher prevalence in prophylactic use than in studies of acute treatment of malaria. However, the low prevalence from mefloquine may have been caused by the inclusion of the MALPRO observational study ^66^ in our meta-analysis, which contained a large sample size, although, as with artemisinin derivatives, no large randomized studies exist on psychiatric effects of mefloquine on healthy subjects.

The commonest domain affected by most drugs was mild neurological perturbations (e.g. dizziness) for malaria studies, and psychiatric or behavioural problems for prophylaxis studies. Mefloquine was associated with impairments in more domains investigated than any other antimalarial drug, probably explaining why it has been commonly mentioned in previous reviews ^87– 89^. Psychiatric manifestations were the most reported outcomes following use of mefloquine from our present study, which is in agreement with previous findings ^75^. Mild neurological perturbations, such as stupor, were largely contributed by use of pyrimethamine (20%), which is still used for prophylaxis of malaria. Given that pyrimethamine is usually combined with sulphadoxine, which had the highest frequency of manifestations in an individual drug category, individuals on these drugs should be monitored closely and patients counselled appropriately. These findings highlight the importance of focusing on assessment of specific domains of mental and neurological manifestations, rather than the overall prevalence with regards to their association with antimalarial drugs.

### Hypotheses of mechanisms of mental and neurological manifestations of antimalarial drugs

Animal studies have indicated that antimalarial drugs commonly affect the hind brain, which contains the reticular formation (controls transitions from sleep to consciousness), and brain stem (has cranial nerves, some innervating the head), perhaps explaining the high prevalence of dizziness and headaches in humans. For instance, mefloquine causes mental and neurological manifestations by disrupting calcium homeostasis of neuronal cells, inhibition of enzymes such as acetylcholinesterase, and blockade of intercellular channels, particularly connexion Cx36, which is a gap junction protein thought to be involved in synchronizing rhythmic activity of neurons in several brain regions ^87^. Chloroquine may interact with multiple neurotransmitter systems: prostaglandin-E antagonism, acetylcholine imbalance and excess dopamine are among the postulated mechanisms ^31^. It is possible that the mechanism of mental and neurological manifestations of antimalarials involves a complex interaction of multiple systems and more studies are required to determine the precise mechanisms by which these deleterious effects occur. The process through which anti-malarial drugs cause damage to the brain is not yet clear, but several hypotheses are proposed for specific drugs, as summarized in Table 8.

**Table 8.** Proposed mechanisms of neurotoxicity.

Drug	Proposed mechanisms of neurotoxicity
Chloroquine	• Sensitize cell-killing effects ^90^ • Cerebrocortical stimulant that increases EEG frequencies ^91^ • Prostaglandin E antagonism ^92^ • Acetylcholinesterase inhibition ^93^ • Depression of cortical activity ^94^ • Inhibition of membrane calcium channels ^95^ • Glucose 6 phosphate dehydrogenase deficiency ^92^ • Alteration of dopamine levels ^93^ • Induction of cholinergic imbalance ^94^
Mefloquine	• Disruption of gap junction communication and GABAergic interneuron dysfunction ^82^ • Inhibition of cellular transport ^87^ • Disrupts direct intercellular electrical communication ^96^ • Acetylcholinesterase inhibition ^42^ • Primary hepatocellular injury ^97^ • Depression of cortical activity ^94^
Minocycline	• Disrupts microglia distribution in the developing somatosensory cortex ^98^ • Modifies electrophysiological properties of layer 5 microglia ^98^
Quinine	• Inhibits cytochrome P450-3A4 ^99^

### Strengths and limitations

The study focused on all types of antimalarial drugs, which provides empirical basis for evaluating new *vs* older drugs and safety profiles of antimalarial drugs recommended by the WHO. We have used robust statistical approaches to estimate the overall prevalence, while accounting for potential heterogeneity between studies. Most of the studies are based on hospital data, which may bias results towards severity, especially for low-income countries where rate of hospital use is often low. We may have underestimated prevalence for specific domains, since some studies did not report multiple domains of mental and neurological manifestations. Our results are based on short-term evaluation of mental and neurological manifestations after antimalarial drugs, so it is unclear what the long-term effects are. Some prevalence estimates are based on a small denominator, thus studies with larger sample sizes are required in future. It is difficult to separate the mental and neurological manifestation of malarial disease from that of the drugs used to treat malaria in settings where drugs were not used for prophylaxis. Some prevalence estimates are based on observational studies rather than randomised controlled studies. As a result, it is difficult to appraise the methodology of observational studies. Additionally our meta-analysis found high between study heterogeneity, which we could not account for in our analysis. Further studies need to be done to explain the differences observed between studies. We applied a stringent quality check on all studies based on GRADE for experimental studies and The Joanna Briggs Institute Prevalence Critical Appraisal Tool for observation studies. This quality check may have excluded a number of children studies.

## Conclusions

This review suggests that mental and neurological manifestations may occur following antimalarial drug use. Potential adverse effects should be assessed and addressed after use of an antimalarial drugs, particularly following prophylactic use. In addition, the mental and neurological effect of antimalarials is poorly researched; few human studies were identified and most of these were not recent. Efforts to develop new effective and safer antimalarial drugs should be accelerated by scientists and development partners. Pharmacovigilance (Phase IV) studies should be set up to document the long-term effects of antimalarials.

## Data availability

The data referenced by this article are under copyright with the following copyright statement: Copyright: © 2017 Bitta MA et al.

The dataset used for this analysis is available on the Open Science Framework: DOI, 10.17605/OSF.IO/2RMCN ( https://osf.io/2rmcn/) ^100^.

## Supplementary materials

**Supplementary File 1: PRISMA checklist.**

**Supplementary Table 1: Search strategy.** We provided the search strategy used in the Pubmed database.

**Supplementary Table 2: Variables for which data were extracted.**
