# Supplementary material for: Antimalarial drugs and the prevalence of mental and neurological manifestations: A systematic review and meta-analysis
Source: Wellcome Open Res. 2017 Jun 2;2:13. Originally published 2017 Feb 20. [Version 2] doi: 10.12688/wellcomeopenres.10658.2 (PMC5473418; doi:10.12688/wellcomeopenres.10658.2)
Supplement: Supplementary file 2 [file wellcomeopenres-2-12747-s0001.tgz › 70e30193-4b80-4d45-8d93-f8a39972c64c.docx]

Supplementary Table 1. Search strategy

| **Search strategy in Pubmed:** |
| --- |
| ((neurotoxicity OR mental health problems OR nervous system changes OR nervous system effects OR adverse neurologic* effects OR neurologic* changes OR neuropathy OR mental disorders OR mental problems OR psychiatric problems OR psychiatric disorders OR encephalopathy* OR nervous system poisoning OR neurotoxic disorders OR neurotoxic problems OR neurotoxin diseases OR nervous system poisoning OR cognitive impairment* OR cognitive deficit* OR neurodevelopmental problems OR neurodevelopmental disorders) AND (antimalarial drugs OR antimalarial agents OR quinine OR chloroquine OR mefloquine OR halofantrine OR artemisinin OR artemether OR artesunate OR proguanil OR pyrimethamine OR sulfalene OR dapsone OR tetracycline OR doxycycline OR minocycline OR primaquine OR artemotil OR arteether OR artenimol OR embonate OR cycloguanil OR pamaquine OR hydroxychloroquine OR amodiaquine OR lumefantrine)) |
