# Supplementary material for: Antimalarial drugs and the prevalence of mental and neurological manifestations: A systematic review and meta-analysis
Source: Wellcome Open Res. 2017 Jun 2;2:13. Originally published 2017 Feb 20. [Version 2] doi: 10.12688/wellcomeopenres.10658.2 (PMC5473418; doi:10.12688/wellcomeopenres.10658.2)
Supplement: Supplementary file 3 [file wellcomeopenres-2-12747-s0002.tgz › cfbc2af6-b8b7-405e-9e71-2e93ed289880.docx]

**Supplementary Table 2. Variables for which data were extracted**

| Data was extracted for the following variables:  Study author, year, country, study design, type of antimalarial drugs, number of antimalarial drugs prescribed, dosage of antimalarial drugs, neurotoxic outcomes, acute malaria status, data on age group and sex, sample size, whether the study was a human or animal study, follow-up time (time between treatment and appearance of first neurotoxic symptoms), severity of the reported symptoms, mechanisms of neurotoxicity and, overall prevalence. |
| --- |
