# Supplementary material for: Antimalarial drugs and the prevalence of mental and neurological manifestations: A systematic review and meta-analysis
Source: Wellcome Open Res. 2017 Jun 2;2:13. Originally published 2017 Feb 20. [Version 2] doi: 10.12688/wellcomeopenres.10658.2 (PMC5473418; doi:10.12688/wellcomeopenres.10658.2)
Supplement: Peer review history

# Peer review: Referee response for version 2

**DOI:** 10.21956/wellcomeopenres.12747.r23173

**Published:** 2017-06-05

**Authors:** Melissa Gladstone

**Affiliations:** 1 Department of Clinical Sciences, Liverpool School of Tropical Medicine, Liverpool, UK; 2 Department of Women and Children's Health, Institute of Translational Medicine, University of Liverpool, Liverpool, UK

I am happy that the authors have addressed the issues in the review and have added specific points to the discussion with regards to the limitations of the study.

I have read this submission. I believe that I have an appropriate level of expertise to confirm that it is of an acceptable scientific standard.

# Peer review: Referee response for version 1

**DOI:** 10.21956/wellcomeopenres.11486.r22085

**Published:** 2017-05-11

**Authors:** Melissa Gladstone

**Affiliations:** 1 Department of Clinical Sciences, Liverpool School of Tropical Medicine, Liverpool, UK; 2 Department of Women and Children's Health, Institute of Translational Medicine, University of Liverpool, Liverpool, UK

This is an excellent systematic review looking at the neuropsychological and neurological side effects of anti malarial medication when provided to children. It provides some evidence through a meta-analysis of the combined evidence for side effects and the differences in side effect profiles between different antimalarial medications. 

 The authors have used really clear rationale for the study and have provided a clear statement of the question that they have. The tables and supplementary files provide most of the data which is required in the PRISMA guidelines. They have very carefully explained the search strategy, the mechanisms for managing the data, the review process and extraction process and the variables assessed. 

 Methodology:

 The authors do mention in their results that the review included all ages of individuals however this was not entirely clear in the methods and might be worth mentioning earlier. It also was not entirely clear which settings in the world the studies were derived from. I assume from the results that the articles were not filtered at all by setting and that therefore they were derived from any setting in the world. This might be important to mention in the methods and in the results.

 The authors clearly delineate the outcomes that they are studying and provide information of how they classified neurological manifestations however it was not clear as to whether the outcomes measured between the different studies were similar in terms of how they measured neuropsychiatric or neurological manifestations, e.g. in children post cerebral malaria vs adult travellers who have been given mefloquine. It might be useful to mention how data in these different studies was gathered and how comparable they were (if not in the methods then at least to discuss it in the discussion section). 

 Many of the studies identified specifically mention neuropsychiatric or neurological manifestations in their results and as far as i can gather, the main aims of these studies were to identify these outcomes. Many other studies must be done to look at the efficacy and comparability of medications for treating malaria where neurological or neuropsychiatric outcomes are an "adverse event" but not necessarily the main crux of the study. Looking back at a review done in children by Ramos-Martin ^1^, there were 15 studies reviewed with children where outcomes relating to ototoxicity were identified. The authors in the present study have only identified 5 studies in children. I was not sure if that was to do with the inclusion criteria  or criteria for quality of studies in this present study or whether it just needed to be clearer that they were only identifying studies where the main aim was to look at neuropsychiatric or neurological outcomes. If so, it might be worth mentioning that in the discussion as a limitation. 

 The authors lump the studies together even though the studies are of very different types, are from different populations (e.g. persons of different age groups, living in different places with different levels of endemicity of malaria, persons who are likely to have some resistance to malaria vs those who do not, different dosages of medications being provided, treatment being for children with cerebral malaria vs adult travellers prophylactically vs symptomatic uncomplicated malaria in children). It might be useful again in the discussion to mention the large heterogeneity in the populations studied which therefore must create some limitations to the data. Despite this there seem to be some trends that emerge that are helpful to share with the scientific community.

I have read this submission. I believe that I have an appropriate level of expertise to confirm that it is of an acceptable scientific standard, however I have significant reservations, as outlined above.

## References

[1] Ramos-MartínVGonzález-MartínezCMackenzieISchmutzhardJPaceCLallooDGTerlouwDJ: Neuroauditory toxicity of artemisinin combination therapies-have safety concerns been addressed?. *Am J Trop Med Hyg*.2014;91(1) : 10.4269/ajtmh.13-0702 62-73 10.4269/ajtmh.13-0702 24865683PMC4080570

# Response

**Published:** 2017-05-29

**Authors:** Mary Bitta

**Affiliations:** KEMRI-Wellcome Trust Research Program, Kenya

Dear Dr Gladstone

 Thank you for reviewing our article.  We have considered your recommendations and addressed them in the manuscript as follows:

  

- We have clarified in the inclusion/exclusion criteria that the eligible studies were not filtered by study settings.
- We have noted in the discussion section that the different methods of assessment of neurological and mental manifestations in individual eligible studies may have contributed to the heterogeneity of pooled estimates observed during the analysis which informed our decision to apply the random effect model rather than the fixed effect model. The random effect model allows the prevalence of mental and neurological manifestations varies from one study to the next.
- We have noted in the limitation section that a quality check on all eligible studies was performed which may have excluded a number of children studies. We already described in the methods section of first version that the quality check was based on the Grading of Recommendations Assessment, Development, and Evaluation (GRADE) system for experimental studies and The Joanna Briggs Institute Prevalence Critical Appraisal Tool for observation studies..
-  We have now noted in the discussion session that the very high between study heterogeneity observed may be partly explained by factors not reported in the included studies such as levels of endemicity of malaria in the study sites and individual factors such as resistance to malaria, as suggested by the reviewer.

# Peer review: Referee response for version 1

**DOI:** 10.21956/wellcomeopenres.11486.r20387

**Published:** 2017-03-29

**Authors:** André Silva-Pinto

**Affiliations:** 1 Infectious Diseases Department, São João Hospital Center (CHSJ), Porto, Portugal

Very good and interesting manuscript. 

- The title is appropriate;
- The abstract is an adequate summary of the article;
- The methods are comprehensive;
- The conclusions are balanced and justified

I have nothing to suggest.

I have read this submission. I believe that I have an appropriate level of expertise to confirm that it is of an acceptable scientific standard.
